# The patient perspective in the era of personalized medicine: What about scanxiety?

**DOI:** 10.1002/cam4.3889

**Published:** 2021-04-09

**Authors:** José A. E. Custers, Lucy Davis, Christina Messiou, Judith B. Prins, Winette T. A. van der Graaf

**Affiliations:** ^1^ Department of Medical Psychology Radboud university medical center Radboud Institute for Health Sciences Nijmegen The Netherlands; ^2^ The Royal Marsden Hospital Foundation Trust and The Institute of Cancer Research London UK; ^3^ Department of Medical Oncology Erasmus MC Cancer Institute, Erasmus University Medical Center Rotterdam The Netherlands; ^4^ Department of Medical Oncology Netherlands Cancer Institute Amsterdam The Netherlands

## Abstract

Frequency of scanning has accelerated in the era of personalized medicine and is related, but not restricted, to the exploding number of clinical trials for new cancer treatments. Particularly in drug trials, but also in clinical practice, patients are followed up by scans frequently, which may vary from every 6 to 12 weeks until progression. The authors aimed to raise awareness for this underreported but widely present “Sword of Damocles” scan‐related issue also referred to as ‘scanxiety.’

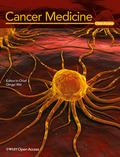

The term “scanxiety” was introduced in 2011 and defined as the anxiety and/or distress associated with an imaging test in postcancer follow‐up, both ahead of the actual examination and up to the announcement of the test results.^1^ In oncology, scanxiety has been described in the context of imaging for assessment of potential metastatic disease in lung cancer^2^ or screening for recurrent disease in long‐term lymphoma survivors.^3^ In these studies, scan‐associated distress was most common in the days and weeks leading up to the examination, and presence of scanxiety was linked with significantly reduced quality of life.^2^ In the postcancer follow‐up setting, imaging tests are scheduled on a regular basis one to four times a year, depending on the risk of cancer recurrence.

Frequency of scanning has accelerated in the era of personalized medicine and is related, but not restricted, to the exploding number of clinical trials for new cancer treatments.^4^, ^5^ Increased understanding of the genetic, molecular, and immunologic basis of cancer and the introduction of personalized medicines, has resulted in the development and introduction of diverse novel therapies and combinations of treatments that did not exist even 5 years ago. Many patients who would once have died within months will now live for years, but with cancer.^6^ Immune checkpoint inhibitors (ICI) were the first class of therapy shown to improve overall survival in patients with advanced melanoma; the anti‐CTLA‐4, anti‐PD‐1, and PDL‐1 antibodies are now part of the standard of care in everyday clinical practice. Moreover, combinations of targeted agents add years of life to those affected by BRAF‐mutated melanomas. Particularly in drug trials,^4^, ^5^ but also in clinical practice patients are followed up by scans frequently, which may vary from every 6 to 12 weeks until progression.^7^, ^8^ However, frequent imaging evaluations of new potentially long‐duration treatments may be distressing for cancer patients, because they typically feel very anxious and uncertain in the weeks before medical check‐ups or consultations, worrying about their medical status and the results. Afterwards, they emotionally process the information and results given in the consultation.^9^

The authors, including a patient, medical oncologist, radiologist, and psychologists met together to raise awareness for this underreported but widely present scan‐related issue in which recurrence or progression of disease continues to hang over patients and their families for the rest of their life like the sword of Damocles. As the patient representative quotes:

“In the weeks running up to the scan I begin to question every single ache and pain and worry that it might mean something. As well as this, the ludicrous number of irrational and often paranoid concerns begin to take over my head. So the pre‐scan nerves are pretty terrifying but as scan day approaches the real fears kick in.”

The scans themselves cause anxiety as patients feel frightened knowing someone is looking for progression. It is also hard to avoid seeking hidden meaning in the manner of the radiology staff wondering what they have seen on the scans. Despite the large numbers of scans which patients undergo, waiting for results gets harder with every scan because it is potentially even more life‐changing/shortening than the last [Patient]. Therewith it is a realistic and justified fear related to possible progression, terminal disease, and being confronted with mortality. The issue of scanxiety should therefore not be addressed as anxiety disorder but relates to the concept of fear of cancer recurrence/progression (FCR), a normal phenomenon for cancer patients. Only a small proportion develops clinical levels of FCR, characterized by high levels of preoccupation, persistent high levels of worry and hypervigilance to bodily symptoms.^10^, ^11^ Whether scanxiety is a distinct feature of FCR, whether it leads to clinical levels of FCR over time or how it is related to the concept of death anxiety^12^ is not yet understood.

This increase in scanning frequency and thus total numbers of scans per patient life might also put extra time pressure on radiologists. The Royal College of Radiologists workforce census revealed a 54% increase in CT scans and 48% increase in MRI scans between 2012 and 2017, which is not matched by an appropriate increase in workforce. As a consequence 8 out of 10 English National Health Service (NHS) trusts have imaging studies unreported for 31 days or more.^13^ Patients trust that if there was anything to worry about, the alarm would be raised but it is possible that radiology review has not yet been undertaken.

“While we wait for results I almost lose the ability to speak, apparently I go a bit grey as those terribly terrifying but very possible scenarios go through my head. Strangely when I do get good results the feeling of relief is remarkably short‐lived. I think it must be too complicated to be able to rejoice in being told that **this time** it’s all ok, **this time**, you’ve got away with it”.

After scan results, patients often have difficulties processing what was talked about in the room with the doctor, and feelings of reassurance are not sustained. Literature shows that 40–80% of medical information provided by healthcare practioners is forgotten immediately, and that almost half of the information remembered is incorrect.^14^, ^15^ Stress and anxiety can lead to attentional narrowing, limiting attentional resources for peripheral information which therefore cannot be recalled.^16^, ^17^ Furthermore, the concept of stable disease remains uncertain as it may mean both tending toward positive or negative results. Healthcare providers most times are aware of the limited recall of information conveyed during consultations and invest in repeated information provision.

The culmination of weeks of pre‐scan nerves, waiting for results and processing of information result in a considerable time period dominated by scan ‐ related fear. Given scan regimes of every 6 to 12 weeks, scanning puts a high burden on patients’ emotional functioning. This raises questions as to who benefits from short scanning intervals? The balance between information on disease status, emotional distress to patients and financial implications for over burdened healthcare systems must be given consideration. Scanning too early during therapy also increases the possibility of non‐conclusive results which exacerbates anxiety related to uncertainty. For patients, increased scanning regimens seem to increase periods of distress. Life prolonging therapies should not only give a patient more days in life, but should also add life to the extra days. Furthermore, ICI trials use imaging protocols that result in high cumulative radiation exposure for patients. Before the ICI era, patients with advanced solid cancer had a very high chance of recurrence or progressive disease and did not live long enough to develop imaging ‐ induced secondary cancer. However, ICI improved the outcomes for a subgroup of patients resulting in increased number of long‐term survivors, who have a higher risk to develop secondary cancer due to imaging radiation.^5^ For oncologists, a broader variety of patient‐centered communication skills and instruments and allocated time for consultations are required. For society, healthcare costs are rapidly increasing and weighing the balance of continuation weeks of ineffective treatments with unnecessary potential side effects should be weighed against the costs and burden of frequent scans.

The results of novel therapies on overall survival are beneficial for some cancer patients with metastatic disease. However, the emotional cost related to medical advances and their development should also be acknowledged and addressed. Fears and hope in metastatic‐cancer survivorship need attention. Defining the precise triggers and causes for scanxiety is an important first step in improving support for patients. For example, the impact of reducing time between scan and result findings should be assessed as a priority and could be a powerful influence on radiology workforce and workflow planning. Rationalizing the imaging schedule in discussion with patients may also alleviate anxieties. It is important to recognise that living with metastatic cancer is stressful and that patients may suffer from this the most outside the walls of the hospital and the immediate scope of their oncologists. Only by exploring with patients what they prefer, can oncology care truly be personalized.

## CONFLICTS OF INTERESTS

The authors declare that they have no conflicts or interests to declare.
